# Role of CD4^+^CD25^hi^CD127^lo/-^FoxP3^+^ regulatory T lymphocytes in the pathogenesis of Behçet’s disease in children

**DOI:** 10.1186/1546-0096-9-S1-P84

**Published:** 2011-09-14

**Authors:** TA Tran, S Monteil, A Letierce, B Terrier, G Geri, D Saadoun, I Kone-Paut, B Salomon, M Rosenzwajg

**Affiliations:** 1 Department of Paediatrics, Pediatric Rheumatology. CEREMAI Bicêtre Hospital, University of Paris Sud., France; 2 Service de Biothérapies/ UPMC CNRS 7211 INSERM 959. La Pitié Salpétrière University Hospital. Paris, France; 3 Unité de Recherche Clinique Paris Sud. Bicêtre University Hospital. Le Kremlin Bicêtre. France; 4 Department of Internal Medicine. La Pitié Salpétrière University Hospital, Paris, France; 5 Unité 2. UPMC-CNRS U7087. La Pitié Salpétrière University Hospital, Paris, France

## Introduction

Behçet’s disease (BD) is an idiopathic multisystem recurrent inflammatory disorder. Physiopathology of BD shows a role of neutrophils and cytotoxic T lymphocytes.

## Our aim

Were to assess the role of regulatory T lymphocytes (Tregs) in the pathogenesis of BD in children.

## Patients and methods

19 patients with active BD (group A) and 8 patients with inactive BD (group B) were compared with 25 healthy controls (group C). Percentages of blood CD4+CD127-CD25hiFoxP3+ Tregs and other T/B and NK cells subpopulations were nalayzed by flow cytometry. The frequency of IL-17A and IFN-γ producing T cells was analyzed by flow cytometer from PBMC after 4 hours stimulation with PMA-ionomycin. We measured serum cytokines by Luminex and ELISA. We compared the 3 groups by using the Wilcoxon-Rank-signed test. Values were expressed as mean and median.

## Results

Patients in the 3 groups (A, B, C respectively) were comparable in term of age and sex distribution (median age: 12.8, 9,9 and 9.7; F/M = 1/1). No differences were observed between the 3 groups concerning the absolute number of lymphocytes, CD4+ T cells and the percentage of total Tregs (median: A: 1.9, B:1.1, C:2.8) . Percentages of naïve Treg/memory Treg and markers of Treg function (GITR, LAP, CD152, DR) were also similar in the 3 groups. However, there was increased CD8+ T cells count in the BD patients groups compared to healthy controls (A: 552±361, p=0.18; B: 627±159, p=0.04, C: 479±209). The NK cell (CD3-CD16+CD56+) were highest in group C compared to group A (p=0.4) or B (p=0.001). IL-17A secreting CD4+ T cells were significantly higher in active BD patients (n=6) compared to controls (n=6) (5.3±2 vs 2.5±1.47, p=0.043). Serum IL-6 level was significantly hisgher in BD populations compared to controls subjects (A: 4,3±1,22 vs C:3±0,7 pg/ml, p=0,016).

## Conclusion

There is no deficit of Tregs number in BD patients. The high rate of peripheral IL-17 secreting CD4+ T cells suggests a possible role of Th17 cells in the occurrence of BD attacks. The Tregs functional ability to regulate CD4 and CD8 T cells needs to be studied further.

